# Ferric Derisomaltose Compared to Iron Sucrose in Iron Deficiency Anemia: A Meta-Analysis of Randomized Controlled Trials

**DOI:** 10.3390/jcm15051919

**Published:** 2026-03-03

**Authors:** Lokman H. Tanriverdi, Ahmet Sarici, Feyzullah Aksan, Adrian V. Hernandez

**Affiliations:** 1 Department of Medical Pharmacology, Faculty of Medicine, İnönü University, Malatya 44100, Türkiye; 2 Division of Hematology, Faculty of Medicine, İnönü University, Malatya 44100, Türkiye; ahmet.sarici@inonu.edu.tr; 3 Department of Internal Medicine, Faculty of Medicine, Stony Brook University, Stony Brook, NY 11794, USA; feyzullah.aksan@stonybrookmedicine.edu; 4 Health Outcomes, Policy, and Evidence Synthesis (HOPES) Group, University of Connecticut School of Pharmacy, Storrs, CT 06269, USA; adrian.hernandez-diaz@uconn.edu; 5 Unidad de Revisiones Sistemáticas y Meta-Análisis (URSIGET), Vicerrectorado de Investigación, Universidad San Ignacio de Loyola (USIL), Lima 15024, Peru

**Keywords:** ferric derisomaltose, iron sucrose, iron deficiency anemia, anaphylaxis, meta-analysis

## Abstract

**Background/Objectives**: There are conflicting results on the effects of ferric derisomaltose (FDM) vs. iron sucrose (IS) for treatment of iron deficiency anemia (IDA). We systematically assessed the efficacy and safety of these treatments. **Methods**: We searched Ovid MEDLINE, Web of Science, Pubmed, and Cochrane Central for randomized controlled trials (RCTs) comparing the efficacy and/or safety of FDM vs. IS in patients with IDA. The primary efficacy outcome was the change in hemoglobin (Hb) levels at week 4, and the safety outcome was serious or severe hypersensitivity reactions, as defined by a standardized set of Medical Dictionary for Regulatory Activities (MedDRA) terms. Inverse-variance random effects models were used for meta-analyses. **Results**: Five RCTs were included: two in general IDA (*n* = 1994), two in non-dialysis chronic kidney disease (*n* = 1542), and one in hemodialysis patients (*n* = 344). The evidence was very uncertain about the effect of FDM vs. IS for Hb change at week 4 (mean difference [MD] 0.09 g/dL, 95% CI −0.33 to 0.52; I^2^ = 84%, very low CoE), serious or severe hypersensitivity reactions (MedDRA A + B + C + D, relative risk [RR] 0.83, 95% CI 0.25 to 2.68; I^2^ = 0%, very low CoE), Hb change at week 8 (MD 0.04 g/dL, 95% CI −0.08 to 0.15; I^2^ = 0%, very low CoE), Hb increase of ≥2 g/dL at week 4 (RR 1.16, 95% CI 0.97 to 1.38; I^2^ = 70%, very low CoE), and anaphylactic reactions (MedDRA A, RR 0.38, 95% CI 0.08 to 1.72; I^2^ = 0%, very low CoE). **Conclusions**: We found that FDM vs. IS had little to no effect on outcomes for the treatment of IDA.

## 1. Introduction

Iron deficiency anemia (IDA) remains a significant global health concern due to its association with increased morbidity and mortality [1,2]. While oral iron supplementation has long been the cornerstone of treatment, its efficacy is often hindered by gastrointestinal side events and lengthy treatment courses [3]. Intravenous (IV) iron preparations offer a promising alternative, particularly for patients with chronic kidney disease (CKD), malabsorption issues, or intolerance to oral iron [4]. The evolution of IV iron therapy has witnessed the emergence of safer and more efficacious formulations, providing clinicians with a wider array of treatment options [5,6].

Historically, the introduction of iron dextran paved the way for IV iron therapy, albeit with significant concerns surrounding anaphylactic reactions. Subsequent innovations led to the development of ferric gluconate and iron sucrose (IS) as safer alternatives, particularly for patients with a history of allergies or intolerance to iron dextran. IS, introduced in 2000, quickly gained traction due to its safety profile and established efficacy, making it a cornerstone of IV iron therapy [7,8,9]. However, the comparison between ferric derisomaltose (FDM, iron isomaltoside) and IS has garnered increasing attention in recent years. While IS is widely used and recognized for its safety profile, its administration requiring multiple infusions and limited weekly dosing may limit its efficacy and patient adherence [10,11,12]. In contrast, FDM, introduced in 2010, has advantages such as rapid high-dose infusion without the need for a test dose and a safety profile as reported in some randomized controlled trials (RCTs); the higher cost of FDM, however, may limit its accessibility to certain patient populations [13,14,15].

The efficacy and safety of these two formulations have been studied in prior RCTs [16,17,18], with conflicting results reported. Derman et al. in 2017 [16] reported that a higher proportion of patients achieved a ≥2 g/dL increase in Hb with FDM compared to IS (68.5% vs. 51.6%), but Auerbach et al. in 2019 [18] reported non-inferiority in Hb change from baseline to week 8 (MD 0.00 mg/dL; 95% CI −0.13 to 0.13) in patients with IDA. On the other hand, Bhandari et al. in 2021 [17] reported that serious or severe hypersensitivity reactions were non-significantly increased in FDM (0.3%) vs. IS (0.0%) in patients with chronic kidney disease (CKD), but they decreased in the study of Auerbach et al. [18] (RD −0.1%; 95% CI, −0.91 to 0.71).

Recent expert consensus guidelines [19] have increasingly emphasized that the choice of intravenous iron formulation should be guided not only by hematologic efficacy but also by dosing efficiency, patient burden, and the risk profile for hypersensitivity reactions. In this context, formulations that allow administration as a single total dose infusion are recommended over those requiring multiple divided doses, as they achieve comparable efficacy while reducing repeated venous access, infusion-related resource utilization, and the cumulative risk of infusion reactions. IS binds elemental iron less tightly to its carbohydrate core, resulting in higher levels of labile free iron and necessitating administration in smaller, repeated doses (typically 200–250 mg per infusion over 4–7 visits) to maintain tolerability [20]. This dosing strategy may limit timely iron repletion and adversely affect adherence. In contrast, FDM exhibits greater complex stability, permitting rapid administration of high single doses (up to 1000 mg or 20 mg/kg) without a test dose, while maintaining a low incidence of serious hypersensitivity reactions. Importantly, the majority of acute infusion reactions with modern IV iron formulations are now recognized as a non–IgE-mediated complement activation–related pseudo-allergy rather than true anaphylaxis, with life-threatening reactions being exceedingly rare [19]. FDM, introduced in Europe in 2009 and approved in the United States in 2020, allows for a total dose infusion of 1000 mg or up to 20 mg/kg in a single session, offering logistical advantages in clinical practice [21]. Within this framework, FDM is classified among the optimal total dose infusion-capable formulations, whereas iron sucrose is considered suboptimal for complete iron repletion in ambulatory patients, despite similar efficacy when cumulative doses are achieved [19].

In this meta-analysis, we compared the efficacy and safety of FDM and IS in the treatment of IDA. Specifically, we evaluated their respective abilities to replenish iron stores and improve hemoglobin levels and their risk of hypersensitivity reactions.

## 2. Materials and Methods

The study followed a prospectively defined protocol that was registered with the International Prospective Register of Systematic Reviews (PROSPERO; CRD42022311652).

### 2.1. Searches

We systematically searched the Cochrane Central Register of Controlled Trials (CENTRAL), Ovid MEDLINE, PubMed, and Web of Science from database inception through 20 May 2024. The search strategy is available in the Supplementary Material. To ensure completeness and currency of the evidence, the literature search was subsequently updated across the same databases to include records published between May 2024 and 1 February 2026; no additional eligible RCTs were identified. Furthermore, the reference lists of included studies, systematic reviews, and meta-analyses from the last five years on comparison of FDM versus IS were reviewed for relevant studies. We limited searches to records published in English.

### 2.2. Study Selection

Two reviewers (LHT and AS) independently searched and reviewed the studies, with disagreements to be solved by a third reviewer (AVH). We included RCTs evaluating FDM vs. IS, conducted in patients with IDA. Studies were excluded if they enrolled participants younger than 18 years, did not report at least one prespecified outcome, or were not published in English.

### 2.3. Outcomes

We used standardized Medical Dictionary for Regulatory Activities (MedDRA) definitions for safety outcomes (Supplementary Table S1). The primary efficacy outcome was change in hemoglobin (Hb) levels at week 4, whereas the primary safety outcome was serious or severe hypersensitivity reactions (MedDRA A + B + C + D). Secondary outcomes included Hb increase of ≥2 g/dL at weeks 4 and 8, Hb change at week 8, and anaphylactic reactions (MedDRA A).

### 2.4. Data Extraction

Data extraction was completed by a single reviewer (LHT) before being then checked by a second reviewer (FA) in a predefined Excel format (Microsoft Corporation, Redmond, WA, USA). Disagreements were resolved with a third reviewer (AVH). Reviewers contacted the principal investigator of the selected RCTs and sponsored company to obtain additional required data from their RCTs. Extracted data included the following: (1) first author; (2) year of publication; (3) etiology of IDA; (4) number of randomized patients; (5) number of final patients; (6) FDM dose and duration; (7) IS dose and duration; (8) time of follow-up in months; (9) primary outcomes per arm; and (10) secondary outcomes per arm.

### 2.5. Risk of Bias Assessment

Two reviewers (LHT, AVH) independently evaluated the risk of bias (RoB) of the RCTs using the Cochrane risk of bias tool RoB2.0 [22], and disagreements were resolved by discussion. The RoB2.0 tool assesses 5 domains of bias: randomization process, deviations from intended interventions, missing outcome data, measurement of the outcome, and selection of the reported result. Judgements of bias per domain can be “low,” “high,” or “some concerns.” The presence of high RoB in at least one domain means the study is at high RoB; the presence of some concerns in at least one domain without a single domain at high RoB means the study has some concerns of bias.

### 2.6. Statistical Analyses

This study was reported in accordance with the Preferred Reporting Items for Systematic Reviews and Meta-Analyses (PRISMA) 2020 statement [23]. Meta-analyses were primarily conducted using inverse-variance random-effects models. For rare outcomes, defined as an incidence below 10%, pooled estimates were calculated using the Mantel–Haenszel approach. Between-study variance (τ^2^) was estimated using the Paule–Mandel method [24], and confidence intervals were adjusted with the Hartung–Knapp method [25] to improve robustness.

Treatment effects for dichotomous outcomes were summarized as relative risks (RRs) and absolute risk differences (ARDs) with corresponding 95% confidence intervals (CIs), whereas continuous outcomes were analyzed using mean differences (MDs) with 95% CIs. Statistical heterogeneity across studies was assessed using Cochran’s Q test and quantified with the I^2^ statistic [26]. I^2^ values of 30–60% were interpreted as moderate heterogeneity, with values exceeding 60% as high heterogeneity and values greater than 75% as substantial heterogeneity.

Prespecified subgroup analyses were performed according to the etiology of iron deficiency anemia and risk of bias. A p value for interaction less than 0.10 was considered indicative of a statistically significant subgroup effect. All analyses were conducted using R software, version 4.1.2 (R Foundation for Statistical Computing, Vienna, Austria).

The certainty of evidence for each outcome was evaluated based on five domains: risk of bias, inconsistency, imprecision, indirectness, and publication bias [27]. Certainty ratings were summarized in summary-of-findings tables generated using GRADEpro software (www.gradepro.org/; McMaster University and Evidence Prime, Hamilton, Ontario, Canada, 2021).

## 3. Results

### 3.1. Selection of Studies

**Figure 1:**
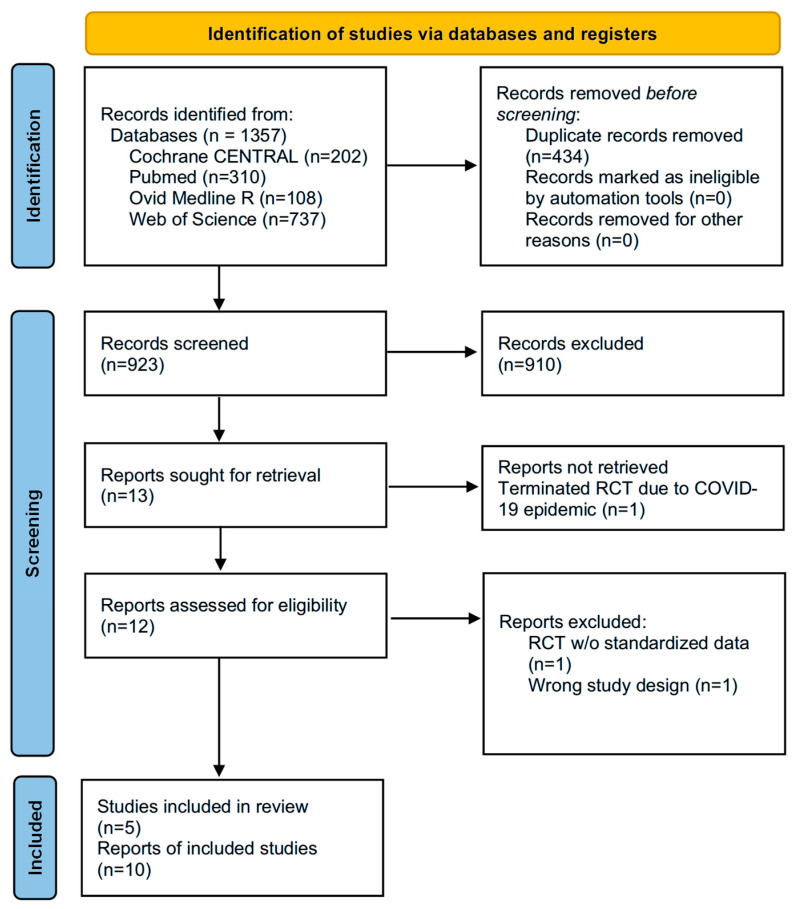
Flowchart of study selection.

The initial search identified a total of 1357 records. After the removal of 434 duplicates, 655 records were screened for eligibility based on title and abstract review. Out of the screened records, 923 studies were excluded for not meeting the inclusion criteria, such as being non-RCTs or lacking comparative data on FDM and IS. The remaining 13 full-text articles were assessed for eligibility. Among these, three reports were excluded for wrong study design (*n* = 1), non-accessible standardized data (*n* = 1) [28], and terminated RCT due to COVID-19 with no publicly reported results available (*n* = 1). Ultimately, five RCTs (*n* = 3952) [14,16,17,18,29] met the eligibility criteria and were included; these corresponded to a total of 10 reports (five primary publications and five registered trial protocols) (Figure 1). Two RCTs [16,18] were conducted in general IDA populations (*n* = 2023), two [17,29] in non-dialysis CKD patients (*n* = 1578), and one [14] in hemodialysis patients (*n* = 351).

### 3.2. Trial Characteristics

Included trials were conducted across multiple countries, including the USA, the UK, India, Russia, Poland, Sweden, Switzerland, Romania, and Denmark (Table 1). Across trials, the average age ranged from 44 to 68.6 years, and the proportion of males varied from 9.8% to 65.8%. Reported outcomes consistently included Hb changes and safety outcomes, such as serious adverse events and hypersensitivity reactions. RCTs varied in their dosing strategies, with FDM generally administered in fewer infusions compared to IS, which required multiple administrations over the study period. Follow-up times ranged from 5 to 13 weeks.

Derman et al., 2017 [16], included 511 participants with multiple-etiology IDA in the USA. The intervention group received a mean cumulative dose of 1640 mg of FDM administered in 1–2 doses, while the control group received 1127 mg of IS over 1–10 administrations. The reported outcomes included hemoglobin (Hb) change at week 4, serious adverse events, and the proportion of patients with an Hb increase of ≥2 g/dL. The follow-up duration was 5 weeks.

Bhandari et al., 2015 [14], evaluated 351 hemodialysis patients with IDA across multiple countries, including India, the UK, Russia, Poland, Sweden, Switzerland, Romania, Denmark, and the USA. The intervention group received a mean dose of 500 mg of FDM over 1–5 administrations, while the control group received 500 mg of IS over three administrations. The primary outcome was the change in Hb levels at week 4, along with the incidence of serious adverse events. Follow-up was conducted for 6 weeks.

Auerbach et al., 2019 [18], studied 1512 patients with multiple-etiology IDA in the USA. The intervention group received a single 975 mg dose of FDM, while the control group received 905 mg of IS administered in five separate doses. The reported outcomes included Hb change at weeks 4 and 8, serious adverse events, and the proportion of patients with an Hb increase of ≥2 g/dL. The follow-up period extended to 8 weeks.

Bhandari et al., 2021 [17], focused on 1538 non-dialysis chronic kidney disease (CKD) patients with IDA in the USA. The intervention consisted of a single 993 mg dose of FDM, while the control group received 899 mg of IS administered over five doses. Outcomes assessed included Hb change at weeks 4 and 8, serious adverse events, and the proportion of patients with an Hb increase of ≥2 g/dL. Follow-up lasted for 8 weeks.

Kassianides et al., 2021 [29], was a smaller trial conducted in the UK with 40 non-dialysis CKD patients. The intervention group received a single 1000 mg dose of FDM, while the control group received a single 200 mg dose of IS. Outcomes included Hb change at week 4 and serious adverse events. The follow-up duration was 13 weeks.

### 3.3. Risk of Bias

For the outcome change in Hb levels at weeks 4 and 8, three trials were rated at high risk of bias due to missing outcome data [14,16,18] and deviations from the intended interventions [14,16]. For hypersensitivity-related adverse events, all five trials had some concerns of bias due to deviations from the intended interventions (Supplementary Figure S1).

### 3.4. Effects of FDM vs. IS on Primary Outcomes

**Figure 2:**
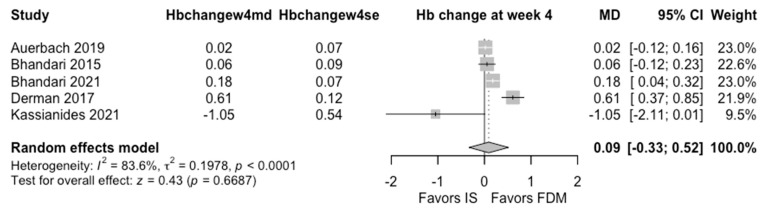
Effects of ferric derisomaltose vs. iron sucrose on Hb change at week 4 [14,16,17,18,29].

**Figure 3:**
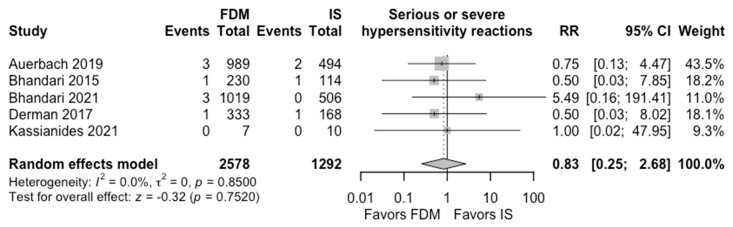
Effects of ferric derisomaltose vs. iron sucrose on serious or severe hypersensitivity reactions (MedDRA: A + B + C + D) [14,16,17,18,29].

The evidence was very uncertain about the effect of FDM vs. IS for Hb change at week 4 (MD 0.09 g/dL, 95% CI: −0.33 to 0.52 g/dL, I^2^ = 84%; 5 RCTs, very low CoE) and serious or severe hypersensitivity reactions (MedDRA: A + B + C + D) (ARD 0.1%, 95% CI: −0.2% to 0.5%; RR 0.83, 95% CI: 0.25 to 2.68, I^2^ = 0%; 5 RCTs, very low CoE) (Table 2, Figure 2 and Figure 3).

### 3.5. Effects of FDM vs. IS on Secondary Outcomes

The evidence was very uncertain about the effect of FDM vs. IS for Hb change at week 8 (MD 0.04 g/dL, 95% CI: −0.08 to 0.15 g/dL, I^2^ = 0%; 2 RCTs, very low CoE); Hb increase of ≥2 g/dL during follow-up (ARD 6.6%, 95% CI: −1.4% to 15.6%; RR 1.14, 95% CI 0.97 to 1.38; I^2^ = 79%, 3 RCTs; very low CoE), at week 4 (ARD 6%, 95% CI: −1.1% to 14.1%; RR 1.16, 95% CI 0.97 to 1.38; I^2^ = 70%, 3 RCTs; very low CoE), and at week 8 (ARD 0.0%, 95% CI: −3.2% to 3.2%; RR 1.00, 95% CI 0.93 to 1.07; I^2^ = 0%, 2 RCTs; very low CoE); and anaphylactic reactions (MedDRA: A) (ARD −0.1, 95% CI: −0.2 to 0.2; RR 0.38, 95% CI 0.08 to 1.72; I^2^ = 0%, 5 RCTs; very low CoE) (Table 2, Supplementary Figures S2–S6).

There were non-significant effects on ferritin (MD 33.15 ng/mL, 95% CI −12.54 to 78.84; I^2^ = 91%, 4 RCTs), iron (MD −1.03 µg/dL, 95% CI −5.54 to 3.48; I^2^ = 59%, 4 RCTs), and transferrin saturation (MD 1.29%, 95% CI −0.89 to 3.46; I^2^ = 85%, 4 RCTs) levels for FDM compared to IS (Supplementary Figures S7–S9).

### 3.6. Subgroup Analyses for Primary Outcomes

Subgroup analyses by IDA etiology and RoB of Hb change at week 4 and serious or severe hypersensitivity reactions (MedDRA A + B + C + D) were consistent with main analyses (Supplementary Figures S10–S13).

## 4. Discussion

Our study suggested that there was little to no effect of FDM vs. IS on Hb improvement and safety profiles in patients with IDA. Certainty of evidence was very low across all primary and secondary outcomes. Subgroup analyses by IDA etiology and RoB for the primary outcomes were consistent with main analyses.

To our knowledge, this study represents the first meta-analysis directly comparing the safety of FDM versus IS in patients with IDA. By systematically synthesizing the available RCTs, it provided the best available evidence on the comparative efficacy and safety of these two intravenous iron formulations. These findings have the potential to guide clinical decision-making and inform future guideline recommendations for the management of IDA, ensuring evidence-based practice in the use of intravenous iron therapies.

A previous meta-analysis by Shi et al. in 2023 [30] compared FDM with IS for the treatment of IDA and included four RCTs comprising 3892 patients. In their quantitative synthesis, FDM and IS demonstrated comparable efficacy with respect to hemoglobin change (SMD = 0.14; 95% CI −0.07 to 0.35; *p* = 0.18), hemoglobin responder rate (SMD = 1.41; 95% CI 0.71 to 2.81; *p* = 0.33), serum ferritin (SMD = 15.13; 95% CI −23.45 to 53.71; *p* = 0.44), and transferrin saturation (SMD = 1.20; 95% CI −1.08 to 3.47; *p* = 0.30). However, a significantly greater short-term increase in serum ferritin at week 2 was observed with FDM compared with IS (SMD = 204.79; 95% CI 38.23 to 371.35; *p* = 0.02), while adverse event rates were similar between groups (OR = 1.11; 95% CI 0.68 to 1.82; *p* = 0.68). Overall, the authors concluded that FDM and IS exhibit broadly comparable efficacy for the management of iron deficiency anemia; however, there were significant methodological differences that impact the reliability of the findings. A major limitation in Shi et al.’s analysis [30] is the exclusion of the Kassianides et al. study [29], indicating a less comprehensive literature search and the potential omission of critical evidence. Their outcome selection, focusing on Hb, ferritin, and transferrin changes specifically at week 2, lacks clinical relevance, as it does not capture the sustained impact of treatment over time. Furthermore, they also failed to define both the duration and level of Hb response, limiting the clinical interpretation of their results [30]. In contrast, our meta-analysis provided a more structured and clinically meaningful approach by specifying exact time points for each outcome, ensuring accurate interpretation of results. Additionally, while the Shi et al. study did not evaluate safety outcomes, our analysis incorporated comprehensive safety measures, including standardized MedDRA definitions, making it a more complete and informative analysis for clinical decision-making.

Kennedy et al. in 2023 [31] conducted a systematic review and meta-analysis focusing on serious or severe hypersensitivity reactions among 10,467 patients from 17 trials (7 with ferric carboxymaltose [FCM] and 10 with FDM). Bayesian inference, naive pooling, and adjusted indirect approaches were used to compare the frequency of hypersensitivity reactions between FDM, FCM, or oral iron by using standardized MedDRA definitions. The incidence of serious hypersensitivity reactions (A + B + C + D) was significantly lower with FDM (5/3474; 0.14%) compared to FCM (29/2683; 1.08%), yielding an odds ratio (OR) of 0.16 (95% CI: 0.05 to 0.33).

Additional evidence from broader safety analyses supports the favorable hypersensitivity profile of FDM. In a large systematic review and meta-analysis including 10,467 patients, Kennedy et al. [31] reported a significantly lower incidence of serious or severe hypersensitivity reactions (MedDRA A + B + C + D) with FDM (5/3474; 0.14%) compared with FCM (29/2683; 1.08%), corresponding to an odds ratio of 0.16 (95% CI 0.05–0.33). Although this analysis did not include a direct comparison with IS, the very low absolute event rate observed with FDM is consistent with the low frequency of serious hypersensitivity reactions identified in the randomized trials included in our meta-analysis. Importantly, our study extends this evidence by providing a direct head-to-head comparison of FDM versus IS using harmonized, standardized MedDRA-coded safety outcomes.

Pollock et al. in 2022 [32] evaluated cardiovascular safety and efficacy among 6042 patients from four RCTs (two comparing FDM with IS and two comparing FCM with IS). Their analysis showed a significantly lower risk of cardiovascular events with FDM compared to IS (OR 0.59; 95% CI: 0.39 to 0.90) and a higher but non-significant risk with FCM (OR 1.12; 95% CI: 0.90 to 1.40). Indirect comparisons further indicated a lower cardiovascular event rate with FDM compared to FCM (OR 0.53; 95% CI: 0.33 to 0.85). However, cardiovascular outcomes were out of the scope of our meta-analysis.

The choice between newer-generation intravenous iron agents such as FDM, FCM, and IS in clinical practice is influenced not only by efficacy and safety considerations but also by cost and administration. FDM permits higher single-dose administration and typically requires fewer infusions than IS, which is administered in multiple smaller doses to achieve equivalent cumulative iron replacement [33]. Although this difference in dosing strategy may improve convenience by reducing infusion frequency and healthcare visits, available evidence—including the findings of the present meta-analysis and our previously published meta-analysis [34] comparing FCM with IS—does not demonstrate a corresponding advantage in hematologic efficacy or safety outcomes. These findings indicate that when equivalent iron doses are administered, newer-generation intravenous iron agents do not confer intrinsic clinical superiority over IS. Consequently, economic considerations, patient preferences, and health system factors remain central to shared decision-making when selecting an intravenous iron formulation for patients with IDA.

### Strengths and Limitations

This meta-analysis has several strengths. The use of the GRADE framework allowed for a systematic assessment of CoE per outcome. Furthermore, the inclusion of diverse populations, including non-dialysis CKD and hemodialysis patients, strengthens the generalizability of the findings across various clinical contexts. A key methodological advantage of this analysis is the incorporation of standardized MedDRA-coded safety data directly obtained from the manufacturer following formal contact with the company. As a result, the present study provides the most comprehensive and methodologically robust synthesis of safety outcomes available on this topic to date.

However, several limitations must be acknowledged. The CoE across all critical outcomes was rated as very low, primarily due to high risk of bias, inconsistency, and imprecision in the included studies. Several trials exhibited methodological limitations, such as incomplete blinding, deviations from intended interventions, and missing outcome data, all of which reduced confidence in the findings. Heterogeneity was another concern, with substantial statistical heterogeneity (I^2^ > 75%) for some outcomes, particularly for Hb change and serious adverse events, indicating variability in treatment effects that could not be fully explained by differences in population or interventions. Another key limitation was the exclusion of the RCT conducted by Kawabata et al. [28], which compared FDM vs. saccharated ferric oxide in IDA associated with menorrhagia that reported non-inferior Hb improvements for FDM vs. saccharated ferric oxide, lower treatment-emergent adverse events (66.2% vs. 90.8%), and significantly reduced hypophosphatemia rates (8.4% vs. 83.2%, severe cases: 0% vs. 6.7%). The decision to exclude this study was due to a lack of standardized data for our safety outcomes comparable to the datasets used in our analysis. Furthermore, the differences in iron formulations and their availability across regions may influence clinical outcomes. The saccharated ferric oxide, primarily available in Japan, has shown a distinct safety profile with higher incidences of hypophosphatemia and related complications such as osteomalacia and fractures [35,36,37] which are not typically observed with IS. This raises concerns about the generalizability of findings from studies using saccharated ferric oxide when compared to IS [38].

## 5. Conclusions

While this meta-analysis study provides important insights into the comparative efficacy and safety of FDM and IS, the very low CoE and methodological concerns warrant cautious interpretation of the findings. The inclusion of standardized MedDRA safety data enhances the reliability of this analysis, making it the most comprehensive evidence synthesis available to date on this topic. Further well-designed, large-scale RCTs with standardized outcome reporting and lower RoB are necessary to strengthen the evidence base for clinical decision-making.

## Figures and Tables

**Table 1:** Characteristics of five included randomized controlled trials.

First Author, Year Reference	Country (Ies)	Population	Sample Size	Intervention, Mean Dose (mg), Number of Administrations	Control, Mean Dose (mg), Number of Administrations	Mean Age (SD), Years	Male (%)	Reported Outcomes	Time of Follow-Up (Weeks)
Bhandari, 2015 [14]	India, UK, Russia, Poland, Sweden, Switzerland, Romania, Denmark, USA	Hemodialysis patients and iron deficiency anemia	351	FDM, 500 mg, 1–5 times	IS, 500 mg, 3 times	59.9 (15.9)	65.8	Change in Hb at week 4, serious adverse events (SMQ)	6
Derman, 2017 [16]	USA	Iron deficiency anemia (multiple etiology)	511	FDM, 1640 mg, 1–2 times	IS, 1127 mg, 1–10 times	48 (16)	9.8	Change in Hb at week 4, serious adverse events (SMQ), 2 g/dL increase in Hb levels at week 4 and during follow-up	5
Auerbach, 2019 [18]	USA	Iron deficiency anemia (multiple etiology)	1512	FDM, 975 mg, 1 time	IS, 905 mg, 5 times	44 (14.7)	10.8	Change in Hb at week 4 and 8, serious adverse events (SMQ), 2 g/dL increase in Hb levels at week 4, week 8, and during follow-up	8
Bhandari, 2021 [17]	USA	Non-dialysis-dependent chronic kidney disease and iron deficiency anemia	1538	FDM, 993 mg, 1 time	IS, 899 mg, 5 times	68.6 (12.3)	37.5	Change in Hb at week 4 and 8, serious adverse events (SMQ), 2 g/dL increase in Hb levels at week 4, week 8, and during follow-up	8
Kassianides, 2021 [29]	UK	Non-dialysis-dependent chronic kidney disease and iron deficiency anemia	40	FDM, 1000 mg, 1 time	IS, 200 mg, 1 time	58.8 (13.9)	57.5	Change in Hb at week 4, serious adverse events	13

Abbreviations: Hb: hemoglobin, FDM: ferric derisomaltose, IS: iron sucrose, SMQ: Standardized Medical Dictionary for Regulatory Activities Queries.

**Table 2:** Summary of findings table of effects of ferric derisomaltose (FDM) vs. iron sucrose (IS) in patients with iron deficiency anemia.

Outcome № of Participants (Studies)	Relative Effect (95% CI)	Anticipated Absolute Effects (95% CI)			Certainty of Evidence
		IS	FDM	Difference	
Change in Hb at week 4 assessed with: Serum Hb measurement follow-up: 4 weeks № of participants: 3588 (5 RCTs)	-	The mean change in Hb at week 4 was **1.38** mg/dL	-	MD **0.09 mg/dL higher** (0.33 lower to 0.52 higher)	⨁◯◯◯ Very low ^a,b,c^
Serious or severe hypersensitivity reaction assessed with: MedDRA definitions A + B + C + D follow-up: range 5 weeks to 13 weeks № of participants: 3870 (5 RCTs)	**RR 0.83** (0.25 to 2.68)	0.3%	**0.3%** (0.1 to 0.8)	**0.1% fewer** (0.2 fewer to 0.5 more)	⨁◯◯◯ Very low ^d,e,f^
Change in Hb at week 8 assessed with: Serum Hb measurement follow-up: 8 weeks № of participants: 2799 (2 RCTs)	-	The mean change in Hb at week 8 was **1.80** mg/dL	-	MD **0.04 mg/dL higher** (0.08 lower to 0.15 higher)	⨁◯◯◯ Very low ^f,g,h^
Hb increase of ≥2 g/dL from baseline to any time during follow-up assessed with: Serum Hb measurement follow-up: range 5 weeks to 13 weeks № of participants: 3540 (3 RCTs)	**RR 1.14** (0.97 to 1.33)	47.4%	**54.0%** (45.9 to 63)	**6.6% more** (1.4 fewer to 15.6 more)	⨁◯◯◯ Very low ^i,j,k^
Hb increase of ≥2 g/dL at week 4 assessed with: Serum Hb measurement follow-up: 4 weeks № of participants: 3244 (3 RCTs)	**RR 1.16** (0.97 to 1.38)	37.2%	**43.2%** (36.1 to 51.3)	**6.0% more** (1.1 fewer to 14.1 more)	⨁◯◯◯ Very low ^j,l,m^
Hb increase of ≥2 g/dL at week 8 assessed with: Serum Hb measurement follow-up: 8 weeks № of participants: 2799 (2 RCTs)	**RR 1.00** (0.93 to 1.07)	46.3%	**46.3%** (43 to 49.5)	**0.0% fewer** (3.2 fewer to 3.2 more)	⨁◯◯◯ Very low ^f,g,n^
Anaphylactic reactions assessed with: MedDRA definitions A follow-up: range 5 weeks to 13 weeks № of participants: 3870 (5 RCTs)	**RR 0.38** (0.08 to 1.72)	0.2%	**0.1%** (0 to 0.4)	**0.1% fewer** (0.2 fewer to 0.2 more)	⨁◯◯◯ Very low ^d,f,o^

**Explanations:** ^a^ Risk of bias (RoB): Downgraded two levels as three RCTs were at high RoB and two RCTs were at low RoB. Derman 2017 [16] and Bhandari 2015 [14] were at high RoB due to deviation for the intended intervention and missing outcome data; Auerbach 2019 [18] was at high RoB due to missing outcome data. Bhandari 2021 [17] had some concerns of bias in the randomization process, deviation from the intended intervention, and selection of the reported result; Kassianides 2021 [29] had some concerns of bias in the deviation from the intended intervention and selection of the reported results. ^b^ Inconsistency: Downgraded two levels due to very high heterogeneity: I^2^ was 84%. ^c^ Imprecision: Downgraded one level as the upper and lower limits of the 95% CI provide effects in opposite direction; 95% CI of the MD was −0.33 to 0.52 mg/dL. ^d^ Risk of bias (RoB): Downgraded two levels as five RCTs were at some concerns of RoB. Derman 2017 [16] was at some concerns of bias in the randomization process, deviation from the intended intervention, measurement of outcome, and selection of the reported result. Auerbach 2019 [18] and Bhandari 2021 [17] had some concerns of bias in the randomization process and deviation from the intended intervention. Kassianides 2021 [29] and Bhandari 2015 [14] had also some concerns of bias due to deviation from the intended intervention, measurement of outcome, and selection of the reported result. ^e^ Imprecision: Downgraded one level due to a small number of events in a few RCTs. The 95% CI around the absolute risk difference (ARD) was narrow (−0.2% to 0.5%). ^f^ Inconsistency: No downgrading as I^2^ was 0%. ^g^ Risk of bias (RoB): Downgraded two levels as one RCT was at high RoB, and one RCT had some concerns of RoB. Auerbach 2019 [18] was at high RoB due to missing outcome data; Bhandari 2021 [17] had some concerns of bias in the randomization process, deviation from the intended intervention, and selection of the reported result. ^h^ Imprecision: Downgraded one level as the upper and lower limits of the 95% CI provide effects in opposite directions; 95% CI of the MD was −0.08 to 0.15 mg/dL. ^i^ Imprecision: Downgraded two levels as the upper and lower limits of the 95% CI of the ARD provided effects in opposite directions (−1.4% to 15.6%), and the CI width was broad. ^j^ Risk of bias (RoB): Downgraded two levels as two RCTs were at high RoB, and one RCT had some concerns of RoB. Derman 2017 [16] and Auerbach 2019 [18] were at high RoB due to missing outcome data; Derman 2017 [16] was at high RoB due to deviation from the intended intervention. Bhandari 2021 [17] had some concerns of bias in the randomization process, deviation from the intended intervention, and selection of the reported result. ^k^ Inconsistency: Downgraded two levels due to very high heterogeneity: I^2^ was 79%. ^l^ Inconsistency: Downgraded one level due to high heterogeneity: I^2^ was 70%. ^m^ Imprecision: Downgraded two levels as the upper and lower limits of the 95% CI of the ARD provided effects in opposite directions (−1.1% to 14.1%), and the CI width was broad. ^n^ Imprecision: Downgraded one level as the upper and lower limits of the 95% CI of the ARD provided effects in opposite directions (−3.2% to 3.2%). ^o^ Imprecision: Downgraded one level due to small number of events; The 95% CI around the ARD was narrow (−0.2% to 0.2%).

## Data Availability

The original contributions presented in this study are included in the article/Supplementary Material. Further inquiries can be directed to the corresponding author.

## Supplementary Materials

The following supporting information can be downloaded at https://www.mdpi.com/article/10.3390/jcm15051919/s1.

## Abbreviations

The following abbreviations are used in this manuscript:

IDA	iron deficiency anemia
IV	intravenous
CKD	chronic kidney disease
FDM	ferric derisomaltose
RCT	randomized controlled trial
TDI	total dose infusion
MedDRA	Medical Dictionary for Regulatory Activities
RoB	risk of bias
PRISMA	Preferred Reporting Items for Systematic Review and Meta-Analysis
RR	relative risks
ARD	absolute risk differences
CI	confidence intervals
CoE	certainty of evidence
