# Supplementary material for: Ferric Derisomaltose Compared to Iron Sucrose in Iron Deficiency Anemia: A Meta-Analysis of Randomized Controlled Trials
Source: J Clin Med. 2026 Mar 3;15(5):1919. doi: 10.3390/jcm15051919 (PMC12985792; doi:10.3390/jcm15051919)
Supplement: Supplementary file 1 [file jcm-15-01919-s001.zip › jcm-4140539-supplementary.pdf]

Ferric derisomaltose compared to iron sucrose in iron deficiency anemia: systematic review and meta-analysis of randomized controlled trials

Tanriverdi LH et al.

Supplementary file

1. Search strategies………………………………………………………………………. 2
2. Supplementary Tables………………………………………………………….……….. 5
2.1. Table S1. Hypersensitivity terms defined by a standardized set of Medical Dictionary for Regulatory Activities (MedDRA)…………………………………………………….. 5
3. Supplementary Figures……………………………………………………………….. …..……..7
3.1. Figure S1: Risk of bias assessment of included RCTs ……………….………………… 7
3.2. Figure S2. Effects of FDM vs. IS on Hb change at week 8…………….……………….. 8
3.3. Figure S3. Effects of FDM vs. IS on Hb increase of ≥2 g/dL at week 4…….………….. 9
3.4. Figure S4. Effects of FDM vs. IS on Hb increase of ≥2 g/dL at week 8.……………… 10
3.5. Figure S5. Effects of FDM vs. IS on Hb increase of ≥2 g/dL during follow-up…...….. 11
3.6. Figure S6. Effects of FDM vs. IS on anaphylactic reactions (MedDRA: A)………….. 12
3.7. Figure S7. Effects of FDM vs. IS on ferritin change at week 4………………...…… 13
3.8. Figure S8. Effects of FDM vs. IS on iron change at week 4.………………………….. 14
3.9. Figure S9. Effects of FDM vs. IS on transferrin change at week 4. …………………..… 15
3.10. Figure S10: Subgroup analyses of Hb change at week 4 by etiology. ..……………...… 16
3.11. Figure S11: Subgroup analyses of Hb change at week 4 by RoB. ………...………..... 17
3.12. Figure S12: Subgroup analyses of serious or severe hypersensitivity reactions by etiology. ………………………………………………………………………………… 18
3.13. Figure S13: Subgroup analyses of serious or severe hypersensitivity reactions by RoB. ……………... 19

1. **Search strategies (Feb 1, 2026)**

Web of Science
#1 (((((((ALL=(iron deficiency anemia)) OR ALL=(anemia)) OR ALL=(iron deficien*)) OR ALL=(iron deplet*)) OR ALL=(anaemia)) OR ALL=(anemic)) OR ALL=(anaemic))
#2 ((((((ALL=(ferric*)) OR ALL=(isomaltose)) OR ALL=(isomaltoside)) OR ALL=(derisomaltose)) OR ALL=(monoferric)) OR ALL=(monofer)) OR ALL=(diafer)
#3 ((((((((((((((ALL=(sucrose)) OR ALL=(saccharated ferric oxide)) OR ALL=(iron sucrose)) OR ALL=(iron saccharate)) OR ALL=(ferric saccharate)) OR ALL=(ferri saccharate)) OR ALL=(iron (iii) hydroxide sucrose complex)) OR ALL=(venofer)) OR ALL=(hippiron)) OR ALL=(ferrisaccharate)) OR ALL=(ferrivenin)) OR ALL=(sucrofer)) OR ALL=(feojectin)) OR ALL=(ferric oxide saccharate)) OR ALL=(sucroferric oxyhydroxide)
#4 #1 AND #2 AND #3

Cochrane Central Register of Controlled Trials (Cochrane CENTRAL) (limited to trials)
#1 MeSH descriptor: [Anemia, Iron-Deficiency] explode all trees
#2 MeSH descriptor: [Anemia] explode all trees
#3 #1 OR #2
#4 (ferric* OR isomaltose OR isomaltoside OR derisomaltose OR Monoferric OR monofer OR diafer):ti,ab,kw
#5 MeSH descriptor: [iron sucrose] explode all trees
#6 ((sucrose OR 'saccharated ferric oxide' OR 'iron sucrose' OR 'iron saccharate' OR 'ferric saccharate' OR 'ferri saccharate' OR 'iron (iii) hydroxide sucrose complex' OR venofer OR hippiron OR ferrisaccharate OR ferrivenin OR sucrofer OR feojectin OR 'ferric oxide saccharate' OR sucroferric oxyhydroxide)):ti,ab,kw
#7 #5 OR #6
#8 (#3 AND #4 AND #7) in Trials

Ovid Medline (R) (Epub Ahead of Print, In-Process, In-Data-Review & Other Non-Indexed Citations and Daily 1946 to Feb 1, 2026)
1 (exp *Anemia/ or Anemia.mp.) or (exp *Anemia, Iron-Deficiency/ or Anemia, Iron-Deficiency.mp.) {Including Related Terms}
2 exp *Anemia/ or Anemia.mp.
3 1 or 2
4 exp Ferric Oxide, Saccharated/ or Ferric Oxide, Saccharated.mp.
5 (exp Ferric Oxide, Saccharated/ or Ferric Oxide, Saccharated.mp.) {Including Related Terms}
6 Sucrose/ or sucrose.mp.
7 saccharated ferric oxide.mp.
8 iron sucrose.mp.
9 iron saccharate.mp.
10 ferric saccharate.mp.
11 iron (iii) hydroxide sucrose complex {Including Related Terms}
12 venofer.mp.
13 hippiron.mp.

14 ferrisaccharate.mp.
15 ferrivenin.mp.
16 ferric oxide saccharate.mp.
17 sucroferric oxyhydroxide.mp.
18 4 or 5 or 6 or 7 or 8 or 9 or 10 or 11 or 12 or 13 or 14 or 15 or 16 or 17
19 derisomaltose.mp.
20 isomaltose.mp.
21 isomaltoside.mp.
22 monoferric.mp.
23 monofer.mp.
24 diafer.mp.
25 19 or 20 or 21 or 22 or 23 or 24
26 3 and 18 and 25

Pubmed

#1 "anemia, iron deficiency"[MeSH Terms] OR "anemia"[Title/Abstract] OR "iron deficiency"[Title/Abstract] OR "anaemia"[Title/Abstract]

#2 "ferric"[Title/Abstract] OR "isomaltose"[Title/Abstract] OR "isomaltoside"[Title/Abstract] OR "derisomaltose"[Title/Abstract] OR "monoferric"[Title/Abstract] OR "monofer"[Title/Abstract] OR "diafer"[Title/Abstract]

#3 "sucrose"[Title/Abstract] OR "saccharated ferric oxide"[Title/Abstract] OR "iron sucrose"[Title/Abstract] OR "iron saccharate"[Title/Abstract] OR "ferric saccharate"[Title/Abstract] OR "ferri saccharate"[Title/Abstract] OR "iron iii hydroxide sucrose complex"[Title/Abstract] OR "venofer"[Title/Abstract] OR "hippiron"[Title/Abstract] OR "ferrisaccharate"[Title/Abstract] OR "ferrivenin"[Title/Abstract] OR "sucrofer"[Title/Abstract] OR "feojectin"[Title/Abstract] OR (("ferric oxide"[Supplementary Concept] OR "ferric oxide"[All Fields]) AND "saccharate"[Title/Abstract]) OR "sucroferric oxyhydroxide"[Title/Abstract]

#4 #1 AND #2 AND #3

2. Supplementary Tables

Table S1. Hypersensitivity terms defined by a standardized set of Medical Dictionary for Regulatory Activities (MedDRA) terms

Group A	Group B	Group C	Group D
Anaphylactic reaction<br>Anaphylactic shock<br>Anaphylactic transfusion reaction<br>Anaphylactoid reaction<br>Anaphylactoid shock<br>Circulatory collapse<br>First use syndrome<br>Kounis syndrome<br>Shock<br>Type I hypersensitivity	Acute respiratory failure<br>Asthma<br>Bronchial oedema<br>Bronchospasm<br>Cardio-respiratory distress<br>Chest discomfort<br>Choking<br>Choking sensation<br>Circumoral oedema<br>Cough<br>Cyanosis<br>Dyspnoea<br>Hyperventilation<br>Laryngeal dyspnoea<br>Laryngeal oedema<br>Laryngospasm<br>Laryngotracheal oedema<br>Mouth swelling<br>Nasal obstruction<br>Oedema mouth<br>Oropharyngeal spasm<br>Oropharyngeal swelling<br>Respiratory arrest<br>Respiratory distress<br>Respiratory failure<br>Reversible airways obstruction<br>Sensation of foreign body<br>Sneezing<br>Stridor<br>Swollen tongue<br>Tachypnoea<br>Throat tightness<br>Tongue oedema<br>Tracheal obstruction	Allergic oedema<br>Angioedema<br>Erythema<br>Eye oedema<br>Eye pruritus<br>Eye swelling<br>Eyelid oedema<br>Face oedema<br>Flushing<br>Generalised erythema<br>Injection site urticaria<br>Lip oedema<br>Lip swelling<br>Ocular hyperaemia<br>Oedema<br>Periorbital oedema<br>Pruritus<br>Pruritus allergic<br>Pruritus generalised<br>Rash<br>Rash erythematous<br>Rash generalised<br>Rash pruritic<br>Skin swelling<br>Swelling<br>Swelling face<br>Urticaria<br>Urticaria papular	Blood pressure decreased<br>Blood pressure diastolic decreased<br>Blood pressure systolic decreased<br>Cardiac arrest<br>Cardio-respiratory arrest<br>Cardiovascular insufficiency<br>Diastolic hypotension<br>Hypotension

	Tracheal oedema<br>Upper airway obstruction<br>Wheezing		

3. Supplementary Figures

Study ID	Experimental	Comparator	Outcome	D1	D2	D3	D4	D5	Overall
Derman 2017	FDM	IS	Serious or severe hypersensitivity reactions	!	!	+	!	!	!
Derman 2017	FDM	IS	Hb change from baseline to week 4	!	-	-	+	!	-
Auerbach 2019	FDM	IS	Serious or severe hypersensitivity reactions	!	!	+	+	+	!
Auerbach 2019	FDM	IS	Hb change from baseline to week 4	!	!	-	+	+	-
Bhandari 2021	FDM	IS	Serious or severe hypersensitivity reactions	!	!	+	+	+	!
Bhandari 2021	FDM	IS	Hb change from baseline to week 4	!	!	+	+	!	!
Kassianides 2021	FDM	IS	Serious or severe hypersensitivity reactions	+	!	+	!	!	!
Kassianides 2021	FDM	IS	Hb change from baseline to week 4	+	!	+	+	!	!
Bhandari 2015	FDM	IS	Serious or severe hypersensitivity reactions	+	!	+	!	!	!
Bhandari 2015	FDM	IS	Hb change from baseline to week 4	+	-	-	!	!	-

+ Low risk
! Some concerns
- High risk

D1 Randomisation process
D2 Deviations from the intended interventions
D3 Missing outcome data
D4 Measurement of the outcome
D5 Selection of the reported result

Figure S1: Risk of bias assessment of included RCTs.

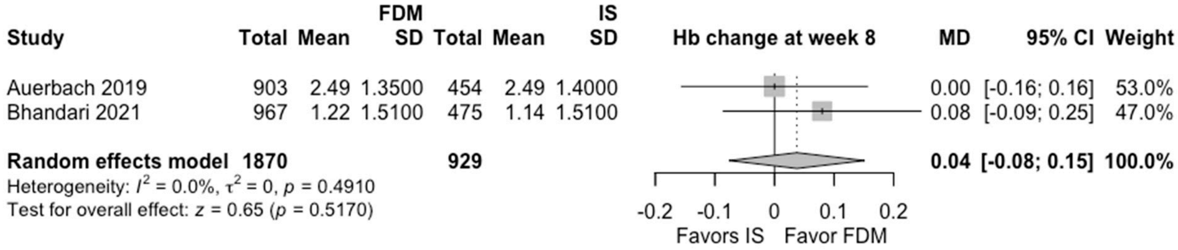

Figure S2. Effects of FDM vs. IS on Hb change at week 8.

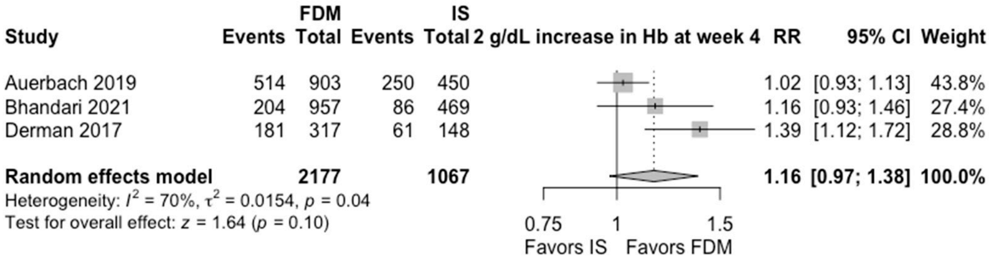

Figure S3. Effects of FDM vs. IS on Hb increase of ≥2 g/dL at week 4.

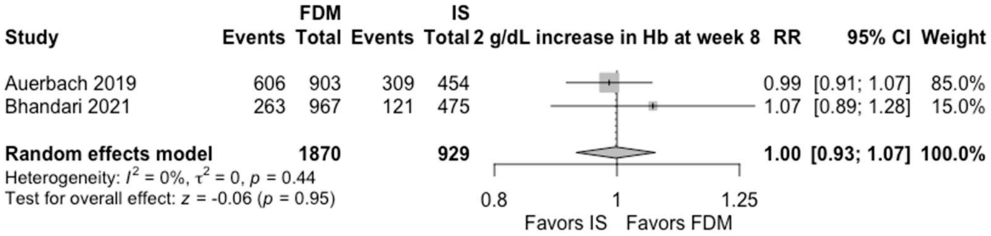

Figure S4. Effects of FDM vs. IS on Hb increase of ≥2 g/dL at week 8.

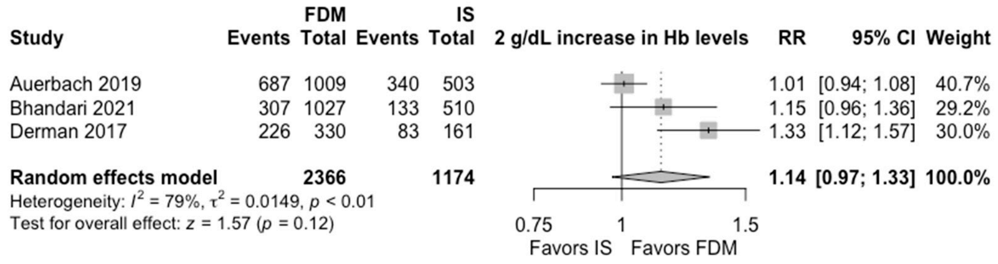

Figure S5. Effects of FDM vs. IS on Hb increase of ≥2 g/dL during follow-up

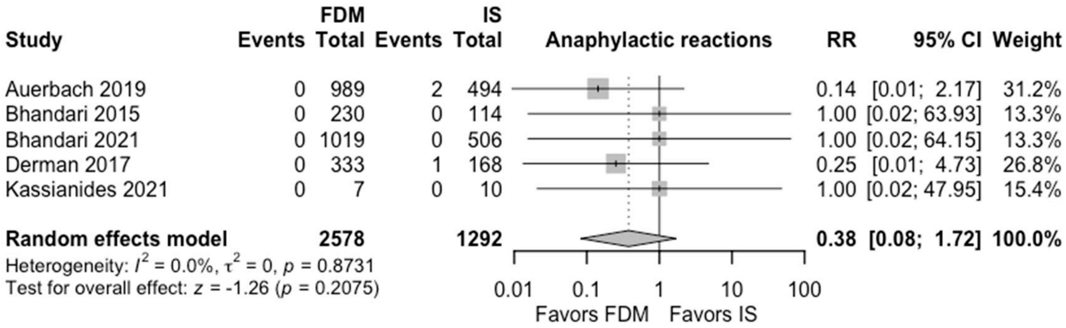

Figure S6. Effects of FDM vs. IS on anaphylactic reactions (MedDRA: A).

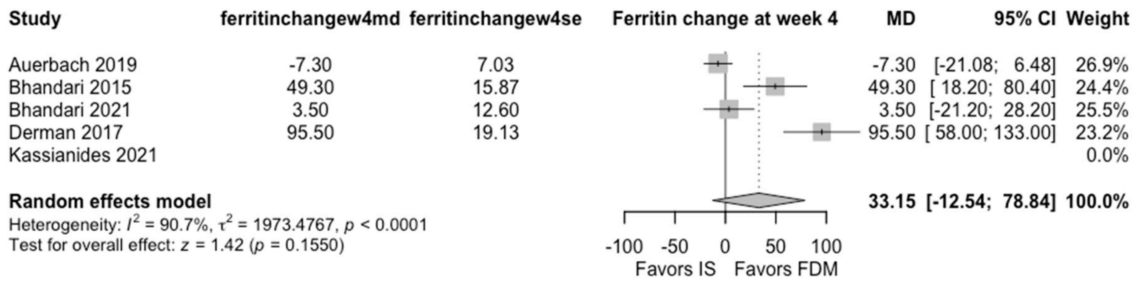

Figure S7. Effects of FDM vs. IS on ferritin change at week 4.

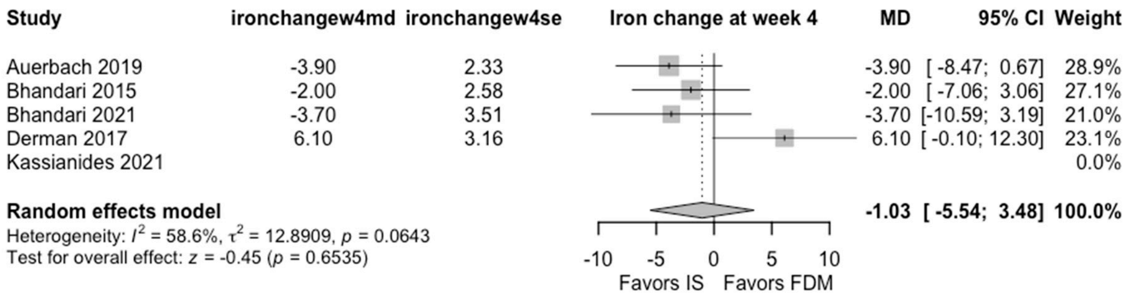

Figure S8. Effects of FDM vs. IS on iron change at week 4.

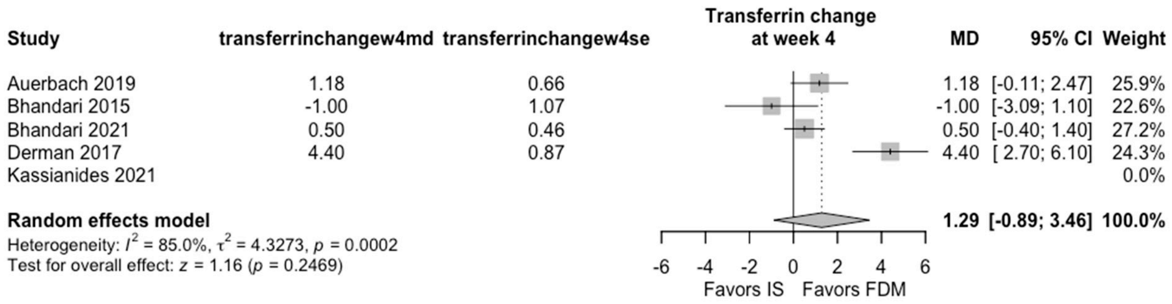

Figure S9. Effects of FDM vs. IS on transferrin change at week 4.

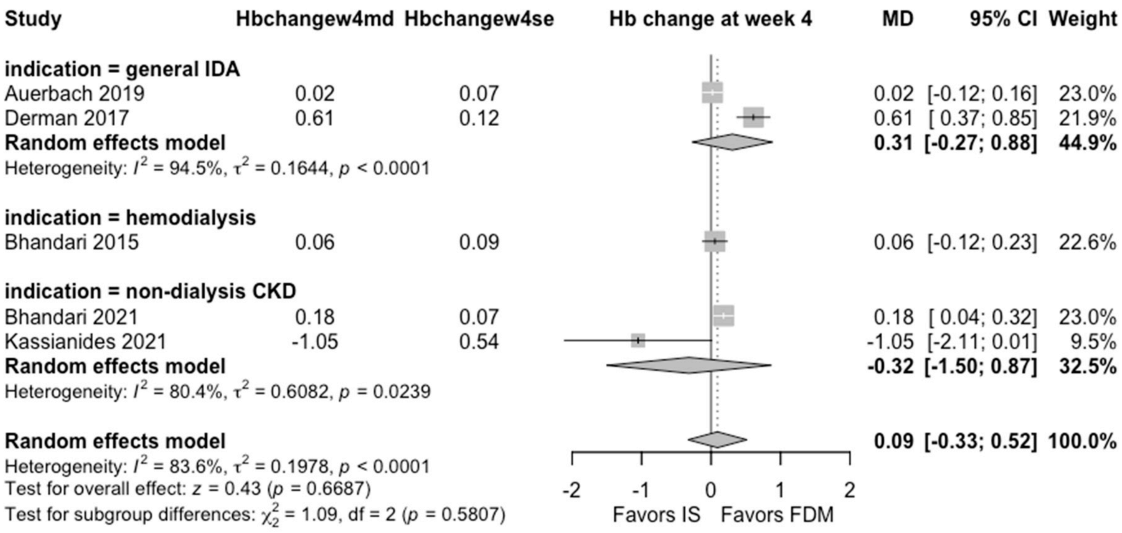

Figure S10: Subgroup analyses of Hb change at week 4 by etiology.

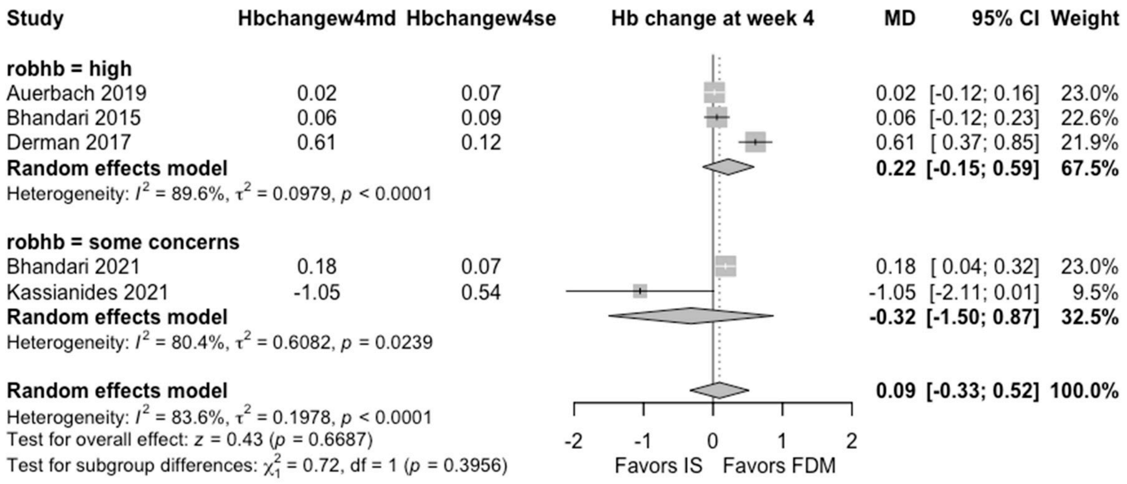

Figure S11: Subgroup analyses of Hb change at week 4 by RoB.

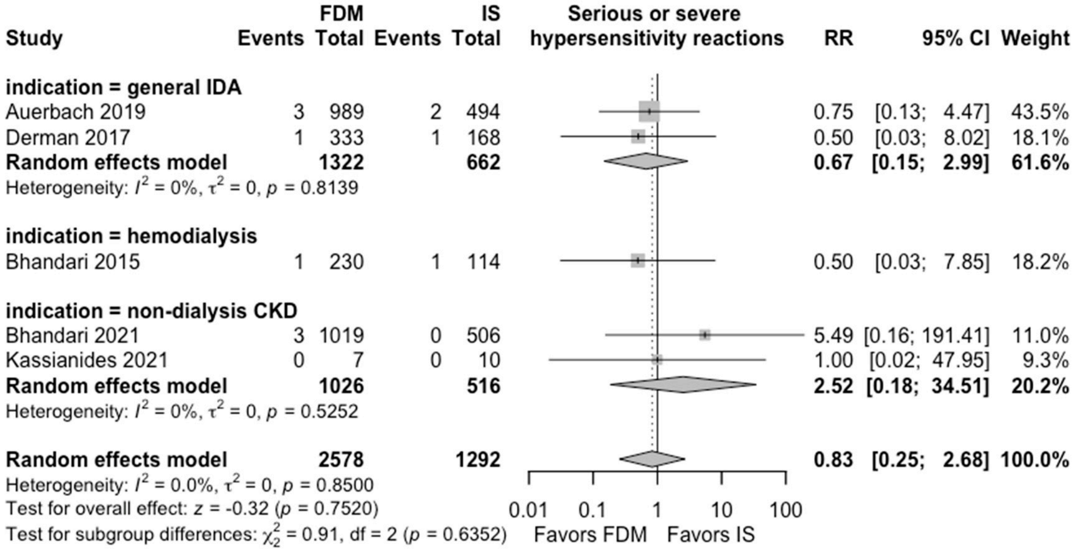

Figure S12: Subgroup analyses of serious or severe hypersensitivity reactions by etiology.

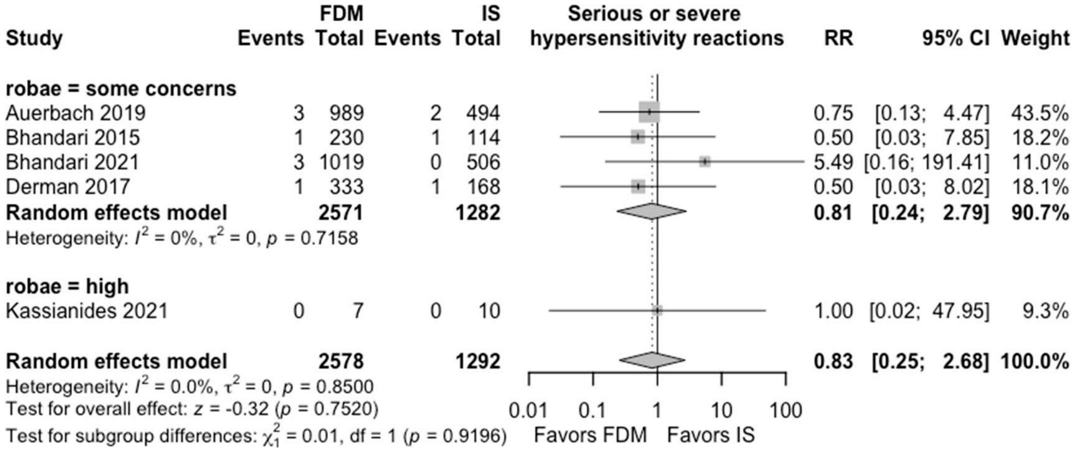

Figure S13: Subgroup analyses of serious or severe hypersensitivity reactions by RoB.
